# Regular Antenatal Care Visits Predict Good Knowledge Among Post-natal Mothers Regarding Entitlements of Health Programs in Western India

**DOI:** 10.15171/ijhpm.2019.28

**Published:** 2019-05-21

**Authors:** Mihir P. Rupani, Pathik M. Patel, Pooja R. Meena, Pooja P. Patel, Priskila A. Patel, Priya K. Paragda

**Affiliations:** Department of Community Medicine, Government Medical College Bhavnagar, Maharaja Krishnakumarsinhji Bhavnagar University, Gujarat, India.

**Keywords:** Antenatal Care, Knowledge, Janani-Shishu Suraksha Karyakram, Janani Suraksha Yojana, Western India

## Abstract

Janani-Shishu Suraksha Karyakram (JSSK) and Janani Suraksha Yojana (JSY) were launched with the objective of increasing institutional deliveries. But, its knowledge among the post-natal mothers is not known. This research evaluated the knowledge of two national health programs among post-natal mothers and found out the predictors of good knowledge about the entitlements of these programs. A cross-sectional study was conducted on a sample of consecutively recruited 339 post-natal mothers who had delivered in a tertiary care hospital of western India. Data were collected from November 2016 to February 2017 by interview method using a questionnaire with questions about knowledge regarding the entitlements of JSSK and JSY. Multivariable analysis was carried out for predictors of good knowledge. Among the 339 post-natal mothers, 30% had a good knowledge regarding JSSK. Only 24% had heard about JSSK; 54% knew regarding free transport to the place of delivery; only 22% and 13%, respectively knew about free inter-facility transport in case of complications for pregnant women and sick infants, while 96% knew regarding free drop-back facility. Only one-fourth of the mothers knew regarding monetary benefit under JSY, while 28% of them had actually received the benefit. The number of antenatal care visits, having an occupation and belonging to Hindu religion significantly predicts good knowledge among postnatal mothers regarding JSSK. Knowledge among the post-natal mothers regarding the entitlements of JSSK and JSY is less while comparing with published literature and needs improvement. Regular ante-natal care (ANC) visits might improve their knowledge of these programs. There is a need to create awareness among hospital staff for the provision of reimbursement of costs incurred by post-natal mothers. There is also a need to carry out demand generation activities among mothers regarding the entitlements of JSSK and JSY.

## Background

The government of India launched the Janani-Shishu Suraksha Karyakram (JSSK) on June 1, 2011 to eliminate out-of-pocket expenditures for both pregnant women and sick neonates.^1^ The initiative entitles all pregnant women delivering in public health institutions to absolutely free and no-expense delivery, including cesarean section. The government of Gujarat extended the benefits of this scheme from neonates to infants until one year of age and post-partum up to 42 days for pregnant women.^2^ A research from Ahmedabad city of Gujarat reported an increase of 20% institutional deliveries after the implementation of JSSK.^3^ Janani Suraksha Yojana (JSY) is a monetary benefit scheme for pregnant women with the objective to increase institutional deliveries.^4^ An amount of 700 Indian rupees is paid 8-12 weeks before delivery to pregnant women who are below poverty line (BPL) or belong to either scheduled caste or scheduled tribe caste.^4^ As per the World Health Organization (WHO) estimates, 5 women died in India every hour during childbirth in 2015.^5^ Maternal mortality ratio of India is estimated as 130 per one lakh live births,^6^ whereas the infant mortality rate of India is estimated as 30 per 1000 live births.^7^ A multi-state study from India highlighted the reduction of maternal mortality ratio through the implementation of JSY program, although also stressed upon provision of quality healthcare services.^8^ Studies from western India reported that 53% of antenatal mothers were aware of the JSY scheme and 46% were aware of free transport service under JSSK among post-natal mothers.^9,10^ A study from Karnataka (south India) reported that lack of knowledge was the major reason for non-utilization of maternity benefit schemes like JSY.^11^ A study from Chhattisgarh (central India) found that parity of the mother was significantly associated with knowledge about JSSK.^12^ Education, nuclear family and advancing age were reported as significant predictors of good knowledge in a study from Maharashtra (western India).^13^ A study from New Delhi (capital of India) reported that belonging to Hindu religion and the number of antenatal care visits were significant predictors of knowledge among post-natal mothers regarding JSY, while the latter was found to be insignificant in predicting knowledge regarding JSSK in West Bengal (eastern India).^14,15^ The predictors of good knowledge of post-natal mothers regarding JSSK and JSY has not yet been evaluated in Gujarat. This research evaluates the knowledge of these programs and attempts to find out predictors of good knowledge regarding these programs among post-natal mothers in Gujarat.

## Methods

### Study Design and Setting

It was a cross-sectional descriptive study conducted in Obstetrics and Gynecology (OBGY) ward of Sir Takhtsinhji Hospital of Bhavnagar in Gujarat state of the western part of India. Sir Takhtsinhji Hospital is a 750-bedded tertiary care government hospital catering to the entire population of Bhavnagar district and is the only tertiary care center for its 11 taluks. It also caters to surrounding districts like Amreli, Junagadh, Gir-Somnath, Surendranagar and Botad districts. Bhavnagar district is located 225 km south-west of Ahmedabad, the financial capital of Gujarat, in the western part of India.

### Inclusion Criteria

The source population is all the post-natal mothers giving birth in Sir Takhtsinhji Hospital of Bhavnagar. The study population consisted of all post-natal mothers who gave birth in Sir Takhtsinhji Hospital at least 2 months back and admitted to OBGY ward for any complaint and giving written informed consent to participate in the study.

### Exclusion Criteria

Post-natal mothers who gave birth within 2 months of the date of data collection or who were seriously ill to unable to respond were excluded from the study. This exclusion was to check whether all the entitlements under JSSK program were received by the beneficiaries or not.

### Sample Size

The sample size of 339 was calculated using Epi Info software version 7,^16^ considering the percentage of institutional births at public health facilities in Bhavnagar as 32.8% as per the National Family Health Survey-4.^17^ The absolute precision was taken as 5% and confidence limits as 95%.

### Study Duration

The study was carried out for a period of 4 months from November 2016 to February 2017.

### Sampling Method and Selection of Subjects

The sampling design was consecutive and data collection was stopped once the required sample size of 339 was achieved. The study participants were interviewed at the OBGY ward of the hospital. In order to maintain the quality of data collection, only three post-natal mothers were interviewed in a day so as to ensure enough time for administering the questionnaire.

### Data Collection Tool

Data collection was done by interview method using a tool prepared based on the guidelines of JSSK of Government of India.^1^ The questionnaire included socio-demographic information; questions on knowledge regarding free transport facility to the place of delivery, inter-facility transfer, drop-back facility, free consumables, and free blood transfusion, etc.^1^ There were nine questions on knowledge regarding various entitlements under JSSK and each were given a score of 1 for knowing and 0 for not knowing. All the scores were added up to create a knowledge score ranging from 0-9. For the purpose of statistical analysis, those post-natal mothers scoring >7 were considered to have good knowledge and those scoring <7 were considered to have poor knowledge. These were author-defined cut-off values based on empirical evidence.

The data on the number of deliveries, 4 years before and after implementation of JSSK in Sir Takhtsinhji hospital (July 1, 2012), was also compared. Questions regarding the awareness and receipt of monetary benefits of JSY were also added.^4^ The questionnaire was pre-tested with a pilot study of 10 post-natal mothers and no changes were found necessary. The data collection was done by the investigators themselves. The principal investigator trained the data collectors on the way of asking questions and cleared any doubts in the questionnaire.

### Variables

The primary outcome variable was a dichotomous variable (good knowledge and poor knowledge regarding JSSK). The predictor variables were the age of the mother in years, years of schooling, parity, possession of BPL card, distance in kilometers of the nearest delivery point, number of ante-natal care **(**ANC) visits, occupation, religion, and caste.

### Statistical Analysis

Data entry and analysis were done in Epi Info software version 7.^16^ Simple proportions were calculated. Multiple logistic regression was carried out to find out significant predictors predicting good knowledge regarding JSSK among post-natal mothers. It was carried out by ‘enter’ method, considering knowledge regarding JSSK as the dependent variable (with 2 outcomes namely good knowledge and poor knowledge) and entering the following variables as the independent variables (predictors predicting knowledge regarding JSSK) in Step 1: age in years, years of schooling, parity, having BPL card, distance in kilometres of nearest delivery point, number of ANC visits, housewife as occupation, Hindu religion and general (open) category. Adjusted odds ratios (OR) with 95% CI were calculated. The difference was said to be significant when the *P* value was <.05.

## Results

A total of 352 post-natal mothers were enrolled in the study out of which 339 participated (response rate of 96.3%).

### Socio-Demographic Information

The mean (± standard deviation [SD]) age of the respondents was 24 (±3.6) years with a range of 14-35 years and their mean (±SD) parity was 2 (±1). The mean (±SD) distance of the nearest delivery center was 22 km (±25). Their mean (±SD) number of ante-natal visits during recent pregnancy was 6 (±3). One-third of the mothers had <4 antenatal care visits during the recent pregnancy and two-thirds of them were BPL cardholders (Table 1). Among the 339 post-natal mothers, 53% belonged to the 20-24 years age group, while 3% were adolescents. About two-thirds of them were literate, with 39% having primary education and 38% being illiterate. Their mean (±SD) years of schooling was 4.5 (±4) years. The majority (84%) belonged to Hindu religion; three-fourths of them were a housewife and 59% belonged to socially and economically backward class.

**Table 1:** Socio-Demographic Profile of the Post-natal Mothers

**Characteristic**	**Total (N = 339)**	**%**
Age group (y)		
<19	10	3
20-24	178	53
25-29	112	33
>30	39	12
Education		
Illiterate	130	38
Just literate	10	3
Primary (5th standard)	133	39
Secondary (10th standard)	47	14
Higher secondary (12th standard)	10	3
Graduate	7	2
Post-graduate	2	1
Literacy status		
Illiterate	130	38
Literate	209	62
Mean (±SD) years of schooling	4.5 (±4.2)	
Religion		
Hindu	285	84
Muslim	48	14
Christian	4	1
Sikh	2	1
Caste		
Socially and economically backward class	200	59
Scheduled caste	61	18
Scheduled tribe	38	11
General (open)	40	12
Occupation		
Housewife	260	77
Laborer	66	19
Tailor	7	2
Health worker	2	1
Diamond worker	2	1
Student	2	1
BPL card holder		
Yes	222	65
No	117	35
Number of ANC visits		
<4	102	30
>4	237	70

Abbreviations: SD, standard deviation; BPL, below poverty line; ANC, ante-natal care.

### Knowledge Among Post-natal Mothers

The median knowledge score among the post-natal mothers regarding JSSK was 5 (range 3-9). Among the 339 post-natal mothers, 30% had a good knowledge regarding JSSK (scoring >7 on the knowledge score). Among the respondents, only 24% had heard about this program; 54% knew regarding free transport to the place of delivery; only 22% knew about free inter-facility transport in case of complications among pregnant women; only 13% knew about free inter-facility transport for sick infants in case of complications, while 96% knew regarding free drop-back facility from hospital to home (Table 2). While 96% of post-natal mothers were offered drop-back facility from this hospital, 6% did not avail this facility as their home was nearby. More than one-third of mothers had paid for transport during the recent delivery, but only 5% of them got reimbursement under JSSK from the hospital.

**Table 2:** Knowledge Regarding JSSK and its Provisions Among the Post-natal Mothers

**Knowledge Questions**	**Total (N = 339)**	**%**
Heard about JSSK		
Yes	83	24
No	256	76
Know regarding free transport to the place of delivery		
Yes	183	54
No	156	46
Know regarding free inter-facility transport for pregnant women		
Yes	76	22
No	263	78
Know regarding free inter-facility transport for sick neonates		
Yes	45	13
No	294	87
Know regarding free drop-back facility from hospital to home		
Yes	326	96
No	13	4
Offered free drop-back facility to home during this delivery		
Yes	326	96
No	13	4
Did not avail free drop-back facility as the home was nearby		
Yes	21	6
No	305	94
Total	326	100
Spent money on transport to the hospital and back home for delivery		
Yes	135	40
No	204	60
Mean (±SD) amount of money paid for transport in Indian rupees	37 (±23)	
Reimbursement of the money spent on transport by the hospital		
Yes	7	5
No	128	95
Total	135	100
Know regarding free investigations, drugs, and consumables		
Yes	327	96
No	12	4
Mean (±SD) amount of money paid for investigations, drugs or consumables during stay at the hospital in Indian rupees	70 (±17)	
Know regarding free food during stay at the hospital		
Yes	338	99.7
No	1	0.3
Offered free food at the hospital		
Yes	326	96
No	13	4
Preferred home food over hospital food		
Yes	53	16
No	286	84
Know regarding free blood transfusion in emergency		
Yes	111	33
No	228	67
Know regarding free user fees (case registration, admission, etc)		
Yes	335	99
No	4	1

Abbreviations: JSSK, Janani-Shishu Suraksha Karyakram; SD, standard deviation.

The average amount of money spent by the mothers on transport and investigations was 37 and 70 Indian rupees respectively. The post-natal mothers did not spend money for food, blood transfusions or any other user fees (case registration charges, admission charges, charges for specialist visits and charges for stay/discharge) at the hospital.

The comparison of the number of deliveries of 4 years before and after implementation of JSSK in the hospital was also analyzed. There was a 29% increase in the number of normal deliveries and a 93% rise in the number of cesarean deliveries after JSSK. The awareness regarding JSY among the mothers was also elicited. Only one-fourth of the mothers knew regarding monetary benefit under JSY, while 28% of them had actually received the benefit. Among those who knew regarding the benefit, 56% did not have a BPL card; 9% did not have at least three antenatal care visits and 7% mothers were not helped by a health worker to get this benefit.

### Predictors of Knowledge Regarding JSSK

The number of ANC visits, being in an occupation other than a housewife and belonging to Hindu religion significantly predicts good knowledge among postnatal mothers regarding JSSK (Table 3). Increase in one ANC visit leads to 1.11 times higher odds of good knowledge regarding JSSK among the post-natal mothers (95% CI: 1.01-1.2). Women who were not housewives were 2 times more likely knowledgeable than women who were housewife (95% CI: 1.04-3.7). Women belonging to Hindu religion were 4 times more likely knowledgeable than women belonging to any other religion (95% CI: 1.2-14.2).

**Table 3:** Adjusted OR of Predictor Variables Predicting Good Knowledge Among Mothers Regarding JSSK by Multiple Logistic Regression^a^ (N = 339)

**Variables**	**Standard Error (Mean)**	**Wald**	**Adjusted OR**	**95% CI**	***P*** **Value**
Age in years	0.053	1.39	1.06	0.96-1.18	.239
Years of schooling	0.036	0.004	0.99	0.93-1.07	.947
Parity	0.205	1.70	0.76	0.51-1.14	.192
Having BPL card	0.310	1.62	1.49	0.81-2.70	.203
Distance in kilometres of nearest delivery point	0.006	0.11	0.99	0.98-1.01	.743
Number of ANC visits	0.047	4.50	1.11	1.01-1.20	.034
Housewife as occupation	0.323	4.30	1.96	1.04-3.70	.038
Hindu religion	0.631	5.03	4.12	1.20-14.20	.025
General (open) category	0.364	0.29	0.82	0.40-1.70	.589
Constant	1.233	4.82	0.07	-	.028

Abbreviations: JSSK, Janani-Shishu Suraksha Karyakram; BPL, below poverty line; ANC, ante-natal care; OR, odds ratio. ^a^Omnibus test of model coefficients *P* <.008; Hosmer Lemeshow test *P* = .468; Nagelkerke R^2^ = 0.099; Classification accuracy 79.1%.

## Discussion

The current study evaluated knowledge among post-natal mothers regarding 2 national health programs – JSSK and YSY – launched with an objective to increase institutional deliveries in Bhavnagar district of western India. The present study also found out predictors predicting good knowledge among post-natal mothers regarding JSSK. JSSK is an initiative of Government of India to assure completely free and cashless services to pregnant women including normal deliveries and cesarean operations (up to 42 days post-partum) and sick infants (up to 1 year after birth) in Government health institutions.^2^ JSY is a safe motherhood intervention under the National Rural Health Mission being implemented with the objective of reducing maternal and neonatal mortality by promoting institutional delivery among the poor pregnant women.^4^ Initially, JSY benefit was paid after delivery before the discharge of the beneficiaries from a health facility.^4^ But since 2009, the monetary benefit of 700 Indian rupees is now given 8-12 weeks before delivery to pregnant women for the purchase of nutritional food.^4^ The benefits of JSY is given only to those pregnant mothers who have a BPL card or belong to scheduled caste/scheduled tribe or have at least three antenatal care visits.^18^

The present study reported that among the post-natal mothers, 30% had good knowledge regarding JSSK. A similar result was reported by a study from West Bengal (eastern India) reporting 31% pregnant mothers having good knowledge.^15^ Another study found only 7% adequate knowledge and 29% moderate knowledge regarding JSSK in Andhra Pradesh (southeast India).^19^ This difference might be due to the fact that the study from southeast India was conducted among ante-natal mothers. A study from Chhattisgarh (central India) reported 59% good knowledge among post-natal mothers regarding JSSK, which was higher than the present study, the difference might be due to a different scoring pattern used in their study.^12^ The lack of knowledge was cited as the commonest reason for non-utilization of maternity benefit schemes in Karnataka (southern India), suggesting the need to create awareness regarding JSSK among ante-natal as well as post-natal mothers.^11^

The present study reported knowledge of 54% among the post-natal mothers regarding free transport to the place of delivery under JSSK. This percentage seems quite less given the fact that in Gujarat, citizens can dial 108 for any kind of emergency, let alone for delivery. This percentage was reported to be even less (28%) in a study in Wardha district of central India, which is a remote district.^20^ A study from eastern India reported 24% of mothers who availed free transport to a health facility and drop-back to home.^21^ A study from Himachal Pradesh (north India) reported 19% of mothers receiving the full benefit for transport.^22^ Only 24% of mothers had even heard of JSSK program in Bhavnagar and the current study reported expenditures on transport and investigations during the period of benefit under this program. A study in the northern part of India reported that the introduction of this program appears to have reduced the out-of-pocket expenditure, but the risk of facing catastrophic health expenditures still remains high.^23^ This might be due to the poor knowledge among pregnant mothers regarding the benefits of this program. The present study highlighted that 40% of beneficiaries spent money on transport during the recent delivery, out of which only 5% of mothers got reimbursement from the hospital under JSSK. Goyal et al reported that 48% of the mothers paid for transport, while none of them got reimbursement from their hospital.^20^ More than half (53%) of mothers had to pay for transport, as reported in a study from West Bengal.^21^ This highlights the need for awareness generation regarding the entitlement of reimbursement of expenses incurred under JSSK program.

The current study stated knowledge of 22% and 13% respectively for free inter-facility transfer for pregnant women and sick infants in case of complications. A similar percentage of 20% was reported in a study in central India for a free transfer to a higher level facility for complications among pregnant women.^20^ The present study highlighted that 96% of mothers knew and availed free drop-back facility from hospital to home post-delivery. This can be attributed to the launch of the ‘Khilkhilat’ Yojana that celebrates the homecoming of the mother and the child after the delivery.^24^ This service was reported to be availed by only 66% of mothers in the study from Wardha district in central India, which is a remote district.^20^

The present study found that almost all the post-natal mothers knew about free user fees and free food during a hospital stay. The present study also found only 33% of mothers being aware of blood transfusion and 96% being aware of free drugs and consumables. The study from West Bengal reported 77% being aware of free admission/stay; 35% about free drugs and consumables; 60% about free food and 29% being aware of free blood for transfusion.^21^ The study from Himachal Pradesh reported that 96%, 86%, and 64% of mothers received free diagnostics, drugs, and consumables respectively.^22^ Thus, the overall knowledge regarding free drugs and consumables seems good in India.

The current study reported that 28% of those who knew regarding JSY received monetary benefits, while a study conducted in West Bengal stated 63% of those eligible receiving the benefits.^21^ A study from another district of West Bengal reported this percentage as 51%.^25^ The researchers in the study from West Bengal also noted that the out-of-pocket expenditure for cesarean delivery could not be covered by cash benefits under JSY.^21^ They further exemplified that the direct cost in a government health facility is mainly contributed by the cost of drugs followed by food and indirect costs by the loss of wages.^21^ The monetary benefits under JSY combined with JSSK were not enough to meet the direct and indirect costs of delivery care.^21^ Evidence from Odisha and Jharkhand found that while the majority of women had heard about JSY (94% in Odisha, 85% in Jharkhand), receipt of benefit was comparatively low (62% in Odisha and 20% in Jharkhand).^26^

On multivariable analysis, the present study found that the number of ANC visits, being in an occupation other than a housewife and belonging to Hindu religion significantly predicted good knowledge among the post-natal mothers regarding JSSK. A study from Maharashtra reported that religion and occupation did not have any significant association with the level of knowledge regarding various entitlements of JSSK in antenatal women.^13^ Another study found that only the distance from referral units was associated with knowledge regarding JSSK and that age, education, occupation, family income, religion, type of family and source of information were not significantly associated.^19^ While a study from western India found that age, monthly income, mother’s education and source of information were significantly associated with knowledge regarding JSSK.^27^ The same study also reported that parity, religion, and type of family were not associated with knowledge regarding JSSK.^27^ Advancing age, mother’s education, nuclear family, gravidity and advancing gestational age were found to be significantly associated with knowledge in another study from western India.^13^ In contrast to the present study, parity of mothers had a statistically significant association and the number of antenatal check-ups did not have a significant association with the knowledge regarding JSSK in a study from West Bengal.^15^ Increasing parity was also found to be associated with good knowledge in the study from Raipur (Chhattisgarh).^12^ The reason for the differences of findings with the present study might be that none of these studies conducted a multivariable analysis for finding out significant predictors after adjusting for confounders. Increasing parity might be increasing their contact with front-line health workers, who might be giving them information, leading to increased knowledge among the mothers. Similarly, as the number of ANC visits increase, there is more possibility of front-line health workers interacting with the pregnant women, counseling them regarding the benefits of institutional delivery and in turn increasing their knowledge regarding the entitlements of JSSK. Being in an occupation other than housewife might lead them to interact more with their peers to gain information regarding the entitlements of JSSK and other benefits in general. Belonging to the Hindu religion might predict good knowledge because of the generally higher female literacy rates and better health-seeking behavior prevalent in this religion. Having more than 6 ANC visits and belonging to Hindu religion were found to be significant predictors among post-natal mothers for availing benefits of JSY in a study conducted in New Delhi.^14^

The study has a few limitations. As this was a cross-sectional study, the causal association between the predictors predicting good knowledge could not be established. The questionnaire and the scoring used in the study was not a validated one, only face validity and content validity of the questionnaire was established. The sampling was consecutive (non-random), as it would have been difficult to obtain a sampling frame in a hospital setting. The sample is not representative of the community and a population-based study would have found even lesser knowledge among the post-natal mothers. The findings are cautiously generalizable to the state of Gujarat, but not to entire India.

In conclusion, knowledge among the post-natal mothers regarding the entitlements of JSSK and JSY in Gujarat is low as compared to other states of India and needs improvement. Regular antenatal care visits, not being a housewife and being a Hindu might predict good knowledge regarding these entitlements among post-natal mothers. There is a need to carry out demand generation activities among mothers regarding the entitlements of JSSK and JSY.

## Acknowledgements

The authors thank Dr. Manindra Pratap Singh, Professor and Head; and Dr. Atul V. Trivedi, Associate Professor of Department of Community Medicine at Government Medical College Bhavnagar, for guiding and supporting us in the study.

## Ethical issues

Written informed consent was taken from the study participants. Ethical approval was taken from the Government Medical College Bhavnagar. Confidentiality of data was maintained by giving unique identifier numbers to each study participant. Personal data was saved in a separate Microsoft Excel sheet which was accessible only to the research team. None of the authors were treating doctors of any of the participants in the study.

## Competing interests

The authors declare that they have no competing interests.

## Authors’ contributions

All the authors contributed to concept, design, definition of intellectual content, literature search, data collection (except first author), field visits for data collection (except first author), data analysis, statistical analysis, manuscript preparation, manuscript editing, and manuscript review. All the authors agree to the final version of this manuscript. The first author will act as the guarantor of this manuscript.
